# Search for Anti-EA(D) Antibodies in Subjects with an “Isolated VCA IgG” Pattern

**DOI:** 10.1155/2010/695104

**Published:** 2010-06-20

**Authors:** Massimo De Paschale, Debora Cagnin, Teresa Cerulli, Maria Teresa Manco, Carlo Agrappi, Paola Mirri, Arianna Gatti, Cristina Rescaldani, Pierangelo Clerici

**Affiliations:** Microbiology Unit, Hospital of Legnano, Via Candiani 2, 20025 Legnano MI, Italy

## Abstract

The presence of an “isolated viral capsid antigen (VCA) IgG” pattern in serum is not easy to interpret without the aid of further tests, such as specific immunoblotting or a virus genome search, that often give rise to organisational and economic problems. However, one alternative is to use an enzyme-linked immunosorbent assay (ELISA) to detect anti-early antigen (EA) antibodies, which can be found in about 85% of subjects with acute Epstein-Barr virus (EBV) infections. The purpose of this work was to search for anti-EA(D) antibodies in 130 samples with an isolated VCA IgG pattern at ELISA screening and classified as being indicative of past (102 cases) or acute (28 cases) infection on the basis of the immunoblotting results. Thirty-seven samples (28.5%) were positive for anti-EA(D), of which 25 (89.3%) had been classified by immunoblotting as indicating acute and 12 (11.8%) past EBV infection. This difference was statistically significant (*P* < .01). The results of our search for anti-EA(D) antibodies correctly identified nearly 90% of acute (presence) or past EBV infections (absence). When other tests are not available, the search for anti-EA antibodies may therefore be helpful in diagnosing patients with an isolated VCA IgG pattern at screening tests.

## 1. Introduction

The most common manifestation of primary Epstein-Barr virus (EBV) infection is acute infectious mononucleosis, a self-limited clinical syndrome that most frequently affects adolescents and young adults. Serology is one of the cardinal means of diagnosing EBV infection as antibody search for viral capsid antigen (VCA), nuclear antigen (EBNA), and early antigen (EA) makes it possible to define the status of the infection [1, 2]. The three parameters of VCA IgG, VCA IgM, and EBNA-1 IgG generally make it simple to distinguish acute and past infection in immunocompetent patients [3]. The presence of VCA IgG and VCA IgM in the absence of EBNA-1 IgG is indicative of acute infection, whereas the presence of VCA IgG and EBNA-1 IgG in the absence of VCA IgM is typical of past infection. 

However, the presence of an isolated VCA IgG pattern may appear in about 8% of all subjects with at least one EBV infection marker [4] and may be difficult to interpret because it can be found in patients with prior infection as well as in those with acute infection. In fact, in some cases, VCA IgM may appear 1-2 weeks after VCA IgG, or for a very short time, or at such a low concentration as to be missed by conventional laboratory tests [5]; furthermore, VCA IgM may persist for a long time after acute infection and still be detected after 80 weeks together with EBNA-1 IgG. The picture is made even more complicated by the fact that 5% of patients produce no EBNA IgG after EBV infection [5, 6] and, even when it is actually produced, it may be lost over time especially in the case of immunosuppression [5, 7, 8]. In such cases, in addition to following up the patient in order to evaluate possible variations in antibody titres, it is useful to perform further laboratory tests such as immunoblotting for various specific IgG antibodies, a VCA IgG avidity test, or searches for heterophile antibodies or viral genome using molecular biology techniques [9]. Tests such as a viral genome search or immunoblotting are particularly useful for defining the status of infection [10–12]. In particular, immunoblotting [13] using recombinant antigens such as p72 (EBNA-1), p18 (VCA), p23 (VCA), p54 (EA), p138 (EA), and gp350/250 (MA = membrane antigen) can detect anti-VCA p18 antibodies which, as they are produced late during the course of EBV infection, are considered substitutes for EBNA-1 IgG [7]. Unfortunately, economic and organisational problems still limit the widespread use of this and molecular biology techniques. 

One possible alternative is to look for an additional serological marker that can be easily detected by means of ELISA cases, such as anti-early antigen (EA) antibodies. These consist of a diffuse (D) and restricted component (R) that reflect the two different patterns originally observed using immunofluorescence. About 70%–85% of patients with acute EBV infection are anti-EA(D) antibody positive for up to three months after symptom onset [7, 14]. However, high titres of these antibodies are present during EBV reactivation [15] and in patients with nasopharyngeal carcinoma [16], and they can also be found in 20%–30% of healthy subjects with a history of EBV infection [17, 18]. Consequently, a search for anti-EA(D) antibodies alone does not make it possible to identify any stage of the disease [9], but its combination with other parameters may be useful for making a laboratory diagnosis of acute EBV infection [19]. A recent study showed a pattern of VCA IgG positive and VCA IgM, EBNA-1 IgG, and anti-EA(D) IgG negative (and heterophile antibody negative) as associated with past infection, while a pattern of VCA IgG and anti-EA(D) IgG positive but VCA IgM and EBNA-1 IgG negative has a still unclear meaning [20].

The aim of this study was to evaluate the usefulness of an ELISA for anti-EA(D) antibodies in subjects with isolated VCA IgG (VCA IgG positive and VCA IgM and EBNA-1 IgG negative) at ELISA screening, typed as being indicative of a past or acute infection on the basis of immunoblotting.

## 2. Material and Methods

One hundred and thirty serum samples were selected with an isolated VCA IgG pattern (VCA IgM and EBNA-1 IgG negative, but VCA IgG positive) at ELISA screening. The samples came from 69 females and 61 males (mean age 32.9 years, range 3–88) with suspected EBV infection and were sent by general practitioners to the Microbiology Unit of Legnano Hospital to be searched for specific antibodies.

In our Unit, ELISA routine screening includes the simultaneous search of VCA IgG, VCA IgM, and EBNA-1 IgG (ETI-VCA-G, ETI-EBV-M reverse, ETI-EBNA-G, DiaSorin, Saluggia, Italy) and in case of isolated VCA IgG, an EBV immunoblotting (RecomBlot EBV IgG, Mikrogen, Neuried, Germany) is performed.

On the basis of the presence or absence of anti-p18 at immunoblotting, all samples were divided into 102 cases with past and 28 with acute infection (Table 1). They were also tested for the presence of heterophile antibodies (MonoSlide, Diesse, Siena, Italy) and anti-EA(D) antibodies, using an ELISA (ETI-EA-G, DiaSorin, Saluggia, Italy). Recombinant polypeptide antigen (47 kDa) used in ELISA is correlated to the recombinant p54 antigen of immunoblotting. The data were statistically analysed using Fisher's exact test and the *χ* ^2^ test.

## 3. Results

Thirty-seven samples (28.5%) were positive and 93 (71.5%) negative for anti-EA(D). Among the cases classified by immunoblotting as indicating acute or past EBV infection, 25 (89.3%) and 12 (11.8%) were respectively positive for anti-EA(D) (Table 2). This difference was statistically significant (*P* < .01). 

Among the 28 cases with acute infection at immunoblotting, 16 (57.1%) had heterophile antibodies and, of these, 15 (93.8%) were positive for anti-EA(D) antibodies.

Table 3 shows the correlation between anti-EA(D) positivity and individual antibody specificity at immunoblotting. The differences were statistically significant in the case of p54, p72, p138, and p18 (*P* < .01).

## 4. Discussion

The presence of an isolated VCA IgG serological pattern is not easy to interpret because it can be found in patients with prior EBV infection who have lost or never shown EBNA-1 IgG as well as in those with acute infection in whom VCA IgM appears late or disappears early. However, it is important to be able to interpret this pattern correctly when a laboratory needs to quickly respond to questions of the clinicians. Without having to wait for a second sample in the hope of a change in the antibody pattern, it would seem to be useful to use a further marker in addition to the three routine tests (VCA-IgG, IgM and EBNA-1 IgG). Given that immunoblotting or a search for virus genome can create organisational and economic problems, an easily automated test such as an ELISA, whose costs are comparable with those of other screening tests, could be a viable alternative. 

Anti-EA(D) antibodies could make a useful marker because they are normally present during the acute phase, even though they are not always produced and sometimes remain for a long time after the primary infection (their presence has been reported in 20%–30% of patients with past infections) [17, 18]. We found them in about 12% of our patients with an isolated VCA IgG pattern and a past infection identified by means of immunoblotting, but in 90% of those with an acute infection. Compared to more recent studies in literature [20], if our study is concordant in indicating, in this group of patients, the absence of anti-EA(D) IgG as mark of past infection, on the other hand the presence of anti-EA(D) IgG correlates with an acute infection.

In conclusion, the results of our search for anti-EA(D) antibodies correctly identified nearly 90% of acute (presence of anti-EA(D)) or past EBV infections (absence of anti-EA(D)), which indicate that, in laboratory routine, it can be helpful in diagnosing immunocompetent patients with an isolated VCA IgG pattern when other more sophisticated tests are not available.

## Figures and Tables

**Table 1:** Antibody specificity at EBV immunoblotting in 103 samples with an isolated VCA IgG pattern at ELISA screening.

No.	Anti-EBV immunoblotting						
	gp250/350	p54	p72	p138	p23	p18	Infection
102	27 (26.5%)	16 (15.7%)	65 (63.7%)	30 (29.4%)	99 (97.1%)	102 (100%)	Past
28	6 (21.4%)	27 (96.4%)	0 (0%)	26 (92.9)	21 (75.0%)	0 (0%)	Acute

**Table 2:** Results of search for anti-EA(D) antibodies in relation to results of anti-EBV immunoblotting.

Anti-EBV immunoblotting	Anti-EA(D) antibodies		
	Negative	Positive	Total
Anti-p18 negative (acute infection)	3 (10.7%)	25 (89.3%)	28
Anti-p18 positive (past infection)	90 (88.2%)	12 (11.8%)	102

**Table 3:** Correlations between the presence of anti-EA(D) antibodies and antibody specificity at EBV immunoblotting.

Anti-EA(D)	No.	Anti-EBV at immunoblotting					
antibodies		gp250/350	p54	p72	p138	p23	p18
Positive	37	11 (29.7%)	34 (91.9%)	7 (18.9%)	32 (86.5%)	32 (86.5%)	12 (32.4%)
Negative	93	22 (23.7%)	13 (14.0%)	58 (62.4%)	24 (25.8%)	89 (95.7%)	92 (98.9%)
*P*		NS	<.01	<.01	<.01	NS	<.01

NS: not significant.
